# Development of Bacteriophage Virus-Like Particle Vaccines Displaying Conserved Epitopes of Dengue Virus Non-Structural Protein 1

**DOI:** 10.3390/vaccines9070726

**Published:** 2021-07-02

**Authors:** Nikole L. Warner, Kathryn M. Frietze

**Affiliations:** 1 Department of Molecular Genetics and Microbiology, School of Medicine, University of New Mexico Health Sciences, Albuquerque, NM 87131, USA; nlwarner@salud.unm.edu; 2 Clinical and Translational Science Center, University of New Mexico Health Sciences, Albuquerque, NM 87131, USA

**Keywords:** dengue virus, non-structural protein 1, vaccine, bacteriophage, virus-like particle, VLP, immunization, ELISA, epitope

## Abstract

Dengue virus (DENV) is a major global health problem, with over half of the world’s population at risk of infection. Despite over 60 years of efforts, no licensed vaccine suitable for population-based immunization against DENV is available. Here, we describe efforts to engineer epitope-based vaccines against DENV non-structural protein 1 (NS1). NS1 is present in DENV-infected cells as well as secreted into the blood of infected individuals. NS1 causes disruption of endothelial cell barriers, resulting in plasma leakage and hemorrhage. Immunizing against NS1 could elicit antibodies that block NS1 function and also target NS1-infected cells for antibody-dependent cell cytotoxicity. We identified highly conserved regions of NS1 from all four DENV serotypes. We generated synthetic peptides to these regions and chemically conjugated them to bacteriophage Qβ virus-like particles (VLPs). Mice were immunized two times with the candidate vaccines and sera were tested for the presence of antibodies that bound to the cognate peptide, recombinant NS1 from all four DENV serotypes, and DENV-2-infected cells. We found that two of the candidate vaccines elicited antibodies that bound to recombinant NS1, and one candidate vaccine elicited antibodies that bound to DENV-infected cells. These results show that an epitope-specific vaccine against conserved regions of NS1 could be a promising approach for DENV vaccines or therapeutics to bind circulating NS1 protein.

## 1. Introduction

Dengue virus (DENV) is an arthropod-borne flavivirus with four serotypes (DENV-1–4) that is transmitted by *Aedes* mosquitos. Every year, approximately 400 million people are infected with dengue virus (DENV), and approximately 10,000 people die as a result of severe DENV disease [1,2,3]. Additionally, 3 billion people reside in areas that are at risk for contracting DENV, yet a reliable and safe vaccine for population-based vaccinations does not exist. The current DENV vaccine, *Dengvaxia* by Sanofi-Pasteur has been criticized for its safety efficacy in population-based vaccination campaigns [4]. This is in part a result of the unique immune response that follows DENV infection. When an individual is infected with any serotype of DENV, that individual can have a range of illnesses from asymptomatic to fever and severe muscle and bone pain, but they develop antibodies against this serotype. Consequentially, if that individual is infected with a heterologous serotype, the individual is at higher risk for developing severe dengue (SD); dengue hemorrhagic fever (DHF) or dengue shock syndrome (DSS) [5,6]. This is likely due to a phenomenon called Antibody-Dependent Enhancement of infection (ADE), whereby non-neutralizing antibodies to structural proteins present on the DENV virion are able to facilitate infection of Fcγ receptor-expressing cells, leading to increased infection and disease. However, if the individual survives the second infection, they make life-long antibodies that protect from all DENV serotype disease in future exposures. This unique antibody response is why protection against all four DENV serotypes must be addressed in a successful vaccine, while avoiding antibodies that can cause ADE.

DENV has a single-stranded, positive-sense RNA genome that encodes three structural proteins, membrane (M), envelope (E), and capsid (C), as well as seven non-structural proteins (NS1, NS2a, NS2b, NS3, NS4a, NS4b, and NS5). Among these non-structural proteins, NS1 has been an attractive target for vaccines. This protein is a 352 amino acid protein that is translated as a monomer during replication of the virus and is glycosylated and forms a dimer in ER of DENV-infected cells [7,8,9,10,11]. This dimeric form is present on both the surface of DENV-infected cells and intracellularly, but NS1 can also be secreted from cells as a hexamer where it can be found circulating in DENV-infected patients. The soluble, hexameric form of NS1 has been associated with inducing vascular leakage of endothelial cells, having a role in autophagy, as well as inducing immune response in peripheral blood mononuclear cells (PBMCs) [12,13,14,15,16]. As such, NS1 has been implicated in playing a role in SD disease. A number of vaccines have been developed against DENV NS1 protein and passive transfer of antibodies against NS1 to naïve mice have been protective in challenge models, suggesting the importance of anti-NS1 antibodies in protection against SD disease [12,17,18,19,20,21,22,23,24,25,26,27,28]. A thorough review of these vaccine strategies and antibody challenge models can be found in the 2018 review by Chen [29]. Targeting DENV NS1 is attractive because antibodies against NS1 should not be at risk of causing ADE because they are not to a structural protein.

Here, we utilized virus-like particles (VLPs) derived from bacteriophage Qβ in order to develop vaccines against DENV NS1. VLP display platforms have been a reliable method for vaccine development, showing success in vaccinations against human papilloma virus (HPV), hepatitis B, and hepatitis E [30]. Additionally, a number of VLP vaccines are currently in human trials for vaccination against viruses such as influenza A, chikungunya, human cytomegalovirus, and human norovirus [30]. Using the self-assembled Qβ bacteriophage, peptides of interest that have a free terminal cysteine can be chemically conjugated to >300 surface lysines available on the self-assembled particle [31,32]. Mice immunized with these peptide-VLP complexes then induce strong antibody responses to the surface peptides [33,34]. Because of the unique nature of immune responses to DENV, we designed our vaccine candidates to target regions of DENV NS1 that were highly conserved among the four DENV serotypes. Here, we utilized Qβ bacteriophage VLPs to immunize mice with 10 rationally designed vaccines and measured the ability of these induced-antibodies to bind to cognate peptides, soluble NS1 from all four DENV serotypes, as well as DENV-infected cells.

## 2. Materials and Methods

### 2.1. Peptide Sequences

The sequences of the entire DENV NS1 protein from all four serotypes (DENV-1–4) were aligned to identify sequence homology and areas of high conservation among the four serotypes. The accession numbers for the reference sequences of each of the four serotypes are as follows: DENV-1 (NP_722461.1), DENV-2 (NP_739584.2), DENV-3 (YP_001531169.2), and DENV-4 (NP_740318.1).

### 2.2. Growth of QB VLPs

Q-Beta (Qβ) VLPs were made using similar methods as previously described [33,35,36]. Briefly, *Escherichia coli* (*E. coli*) C41 cells (Sigma-Aldrich, St. Louis, MO, USA) were transfected with the plasmid pET containing the Qβ coat protein coding sequence under an IPTG-inducible promoter [31,32]. Cells were grown until OD_600_ of 0.6 was reached. Cultures were then induced with 0.5 mM of Isopropyl β-d-1-thiogalactopyranosid (IPTG) for three hours and pelleted by centrifugation. Pellets were resuspended in a lysis buffer consisting of 50 mM Tris-HCl, 10 mM EDTA, and 100 mM NaCl. Cells were then incubated for 30 min on ice after the addition of deoxycholate (DOC) to a final concentration of 0.05%. Suspensions were sonicated for one minute intervals, five times and replaced on ice between sonication. 10 mg/mL of DNase and 2 mM MgCl_2_ was added to the solution and incubated at 37 °C for 1 h to digest residual bacterial DNA. Lysates were centrifuged and ammonium sulfate was added to the supernatant at a 60% saturation overnight. Ammonium sulfate/lysates were centrifuged at 10,000 RPM and pellets were resuspended in sepharose column buffer (SCB) containing 10 mM Tris-HCl, 0.1 M NaCl, and 2 mM MgSO_4_ to a QS of 1 L deionized water. Suspensions were frozen at −80 °C until size-exclusion chromatography was performed. Samples were added to a chromatography column filled with Sepharose CL-48 beads (Sigma-Aldrich) in SCB and fractions containing Qβ VLPs were then identified via agarose gel electrophoresis and denaturing SDS-PAGE gels. Fractions were combined and Qβ VLPs were precipitated by adding 70% ammonium sulfate overnight. A buffer exchange was performed overnight using Snakeskin Dialysis Tubing 10 K molecular weight cutoff (Thermo Fisher Scientific, Waltham, MA, USA) in phosphate buffered saline.

Qβ VLP stocks were then depleted of LPS using sequential Triton X-114 phase extraction. Triton X-114 was added to stocks at a final volume of 1%. Samples were vortexed, incubated on ice for five minutes, followed by a five minute incubation in a 37 °C heat block. Samples were spun at max speed for 1 min at 37 °C. The aqueous phase was moved to a clean tube, and the process was repeated four more times for a total of five times. Concentrations of Qβ was determined by SDS-PAGE gel using known concentrations of hen’s egg lysozyme as a comparison. Correct assembly of Qβ VLPs before and after is assured by buffer exchange and filtration through appropriate molecular weight cutoff such that non-assembled coat protein will not be present in final inoculum. Stocks were frozen at −20 °C until use.

### 2.3. Conjugation of Peptides to Qβ VLPs

Peptides of interest were commercially synthesized by Genscript and reconstituted in the manufacturer’s recommended solvent prior to use. Using the bifunctional crosslinker succinimidyl 6-((beta-maleimidopropionamido) hexanoate) (SMPH), peptides of interest were conjugated to the surface-exposed lysines of Qβ, through an added linker sequence of -GGGC at the C-terminal region. Peptides were added at a 10:1 ratio of peptide to Qβ stock. Excess SMPH and peptide were removed through Amicon filtration (Millipore Sigma, St. Louis, MO, USA).

### 2.4. Immunizations

6–8-week-old BALB/c mice (Jackson Labs, male and female) were vaccinated intramuscularly in the hind leg twice, at three-week intervals, with 5 μg/50 μL of vaccine with no exogenous adjuvant. Retro-orbital bleeds were performed three weeks after first immunization (D21) and three weeks after second immunization (D42) to collect sera for ELISAs. Animals were sacrificed upon verification of antibody titers via cardiac puncture. All animal studies were performed in accordance with guidelines of the University of New Mexico Animal Care and Use Committee (Protocol #: 20-201021-HSC).

### 2.5. ELISA

*Cognate peptides:* Synthetic peptide ELISA was performed as previously described [37]. Briefly, Immunolon 96-well ELISA plates were coated with 1 μg/100 μL of Streptavidin (Invitrogen, St. Louis, MO, USA) in PBS at 4°C overnight. Plates were washed three times with PBS and incubated for one hour at room temp with 2 μg/100 μL of SMPH. Plates were washed with PBS three times and peptides correlating with immunizations were plated in 100 μL volumes at 0.02 μg/μL and incubated for two hours at room temperature. Plates were washed three times with PBS and blocked over night with 100 μL of 0.5% milk in PBS. Plates were washed twice, and mouse sera were diluted in 0.5% milk/PBS in four-fold dilutions starting with 1:40 and ending with 1:655,360. Plates were washed five times and goat anti-mouse conjugated with horseradish peroxidase (HRP) secondary antibody (Jackson ImmunoResearch, Ely, UK) was added at 1:5000 dilution in 50 μL volumes to each well for 45 min. After washing plates 5 times, 50 μL of soluble TMB (Millipore Corp., Burlington, MA, USA) was added to each well. Plates were incubated for 15 min and quenched with 50 μL of 1% HCl solution. Absorbance at 450 nm was determined.

*Soluble DENV NS1:* ELISA against DENV NS1 was performed as follows. A volume of 0.16 μg/well of soluble NS1 protein produced in HEK 293 cells (The Native Antigen Company, Oxford, UK) in 50 μL was added to Immunolon 96-well ELISA plates overnight. Plates were washed three times with PBS and blocked for 2 h at room temperature with 100 μL of 0.5% milk in PBS. Plates were washed 3 times and sera from immunized mice were added in 50 μL volumes and serially diluted starting with a 1:40 dilution and 4-fold dilutions were tested up to 1:655,360. Plates were incubated for 2 h at room temperature with rocking, followed by PBS wash of 5 times. A volume of 50 μL/well of 1:5000 dilution of goat anti-mouse secondary antibody conjugated with HRP (Jackson ImmunoResearch) was added to each well. Plates were washed 5 times with PBS, followed by the addition of 50 μL of TMB for 15 min, and quenched with 50 μL of 1% HCl. Absorbance at 450 nm was determined.

### 2.6. Cell Culture and Virus Stocks

Human embryonic kidney 293 (HEK 293) cells were purchased from American Type Culture Collection (ATCC, CRL-1573). Cells were grown in complete growth medium consisting of ATCC-formulated Eagle’s Minimum Essential Medium (EMEM, cat no. 30-2003) supplemented with a final concentration of 10% fetal bovine serum (FBS).

DENV-2 New Guinea C (NGC), kindly provided by Dr. Kathryn Hanley at New Mexico State University (NMSU), was cultured in C6/36 cells (ATCC) to produce working viral stocks. Virus was collected in 1X SPG consisting of 2.18 M sucrose, 38 mM potassium phosphate (monobasic), 72 mM potassium phosphate (dibasic), and 60 mM L-glutamic acid. Samples were clarified by centrifugation and stored at −80 °C.

### 2.7. Antibody Binding to DENV-Infected Cells

HEK293 cells were plated in 96-well glass bottom plates (Cellvis, Mountain View, CA, USA) at 25,000 cells per well overnight. Cells were washed once with complete HEK media. Cells were infected at an MOI of 100 PFU/well with DENV-2 NGC diluted in complete HEK media for 20 min to allow for binding to cells. 150 μL of HEK media was added to wells and plates were incubated for 72 h at 37°C. Cells were washed 1 time with PBS and fixed two times for 20 min using 200 μL of 100% methanol. Methanol was removed and cells were washed 2 times with PBS and blocked with 100 μL of 2% goat sera (Jackson ImmunoResearch) in PBS for 1 hour at room temperature. Mouse sera were diluted in 2% goat sera/PBS containing a 1:1000 dilution of rabbit anti-DENV 4G2 antibody (The Native Antigen Company). As a positive control, some cells were instead stained with anti-DENV NS1 antibody (mouse monocloncal, Flavivirus NS1 (D/2/D6/B7) (Abcam, Cambridge, UK, 214,337) at a 1:500 dilution. Cells were treated with 50 μL volumes at two-fold dilutions of sera starting with 1:40 going up to 1:320 for 1 hour at room temperature with rocking. Plates were washed three times with PBS. Plates were incubated with 50 μL of Alexa fluor 488 goat anti-mouse IgG (Abcam; 1:1600), Alexa fluor 647 goat anti-rabbit IgG (Jackson ImmunoResearcher; 1:1000), and Hoechst (Thermo-Fisher; 1:2000) for 1 hour at room temperature. Plates were washed 3 times and 150 μL of PBS was replenished to each well. This research made use of the Fluorescence Microscopy and Cell Imaging Shared Resources which is partially supported by UNM Comprehensive Cancer Center Support Grant. Plates were imaged on a Zeiss Axio Observer epifluorescence microscope with a Hamamatsu Flash 4.0 camera using Slidebook imaging software.

## 3. Results

### 3.1. Identification of Conserved Epitopes of NS1 to Target with Vaccination

**Figure 1:**
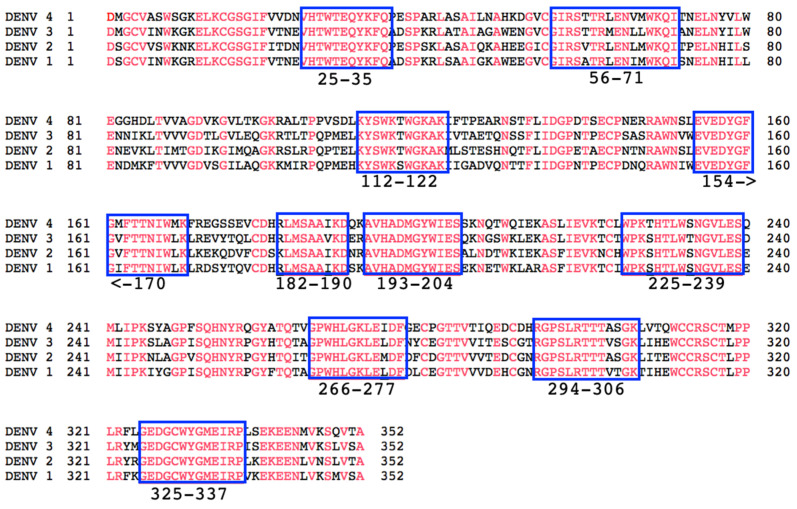
Conservation of NS1 proteins from four DENV serotypes. The amino acid sequence of the four DENV serotypes (Accession #s: DENV-1 (NP_722461.1), DENV-2 (NP_739584.2), DENV-3 (YP_001531169.2), and DENV-4 (NP_740318.1)) were aligned and perfectly conserved amino acids are shown in red. Blue squares indicate the highly conserved regions chosen for investigation as epitope-based vaccine candidates against DENV-NS1, with numbers designating amino acid numbers.

We first aligned the DENV NS1 amino acid sequence of all four DENV serotypes in order to identify highly conserved 9–17 amino acid regions of the NS1 proteins (Figure 1). Our goal is to develop a vaccine candidate that is highly conserved and protective among the four serotypes of DENV. Although most of the vaccine candidates were of highly conserved regions of the NS1 protein where no differences in amino acid sequences were observed (red), we used the sequences from either DENV-2 or DENV-3 for each vaccine candidate. Among the highly conserved regions of NS1 were several regions of interest. The first was aa 112–122 which is the immunodominant region of NS1 wing domain [18,38]. This region of NS1 has been highly associated with antibodies that strongly bind to NS1, but can also have cross-reactive effects in binding to the surface of endothelial cells and inducing damage to these cells [39,40,41,42,43,44,45,46,47,48]. Another region of interest was that of aa 325–337. DENV-infected patients make antibodies to this region of NS1, and these antibodies have been implicated in the binding of platelets and impacting coagulation pathways [47,49]. Interestingly, we have identified antibodies against a number of other highly conserved sequences selected for this study in DENV-infected patient sera including aa 56–71, 154–170, and 193–204 using a deep-sequencing method [50]. Identification of antibodies against these regions of NS1, along with the highly conserved sequences among the four serotypes, gives precedent for the importance of these regions in protecting against DENV disease.

### 3.2. Predicted Surface Exposure of Conserved Regions of DENV NS1 Protein

**Figure 2:**
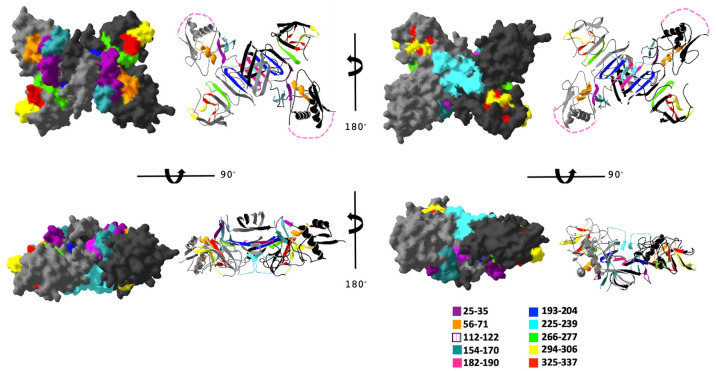
Conserved peptides mapped to the structure of DENV-2 NS1 protein dimer. The DENV-2 NS1 dimer PDB file was imported into Cn3D structure visualization program with each peptide colored as indicated. Different views are shown with space-fill and ribbon structures. Pink dashed lines indicate the assumed location of conserved peptide 112–122, which corresponds to an unresolved disordered region of the NS1 protein.

In order to investigate the potential for each of the conserved DENV NS1 regions to be surface exposed on the protein, we mapped each peptide to the previously published structure of the DENV-2 dimer (PDB ID 4O6B) [51]. The location of each peptide is shown mapped to DENV-2 in Figure 2 (individual peptides are in Supplemental Figure S1). Peptides 25–35, 56–71, 112–122, 154–170, 225–239, 294–306, 325–337 are all at least partially surface exposed in the dimer structure, while peptides 182–190, 193–204, and 266–277 are mostly hidden within the structure.

### 3.3. Qβ VLPs Displaying NS1 Peptides Are Immunogenic in Mice without Exogenous Adjuvant

**Figure 3:**
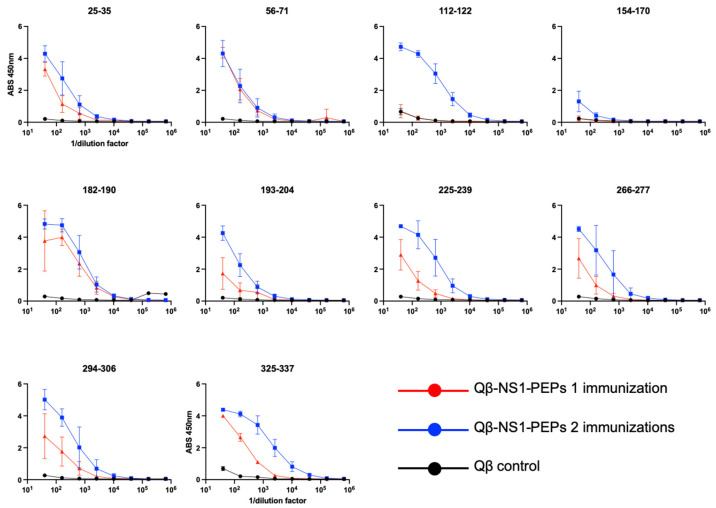
Qβ VLPs displaying NS1 peptides are immunogenic in mice. Mice (*n* = 5/group) were immunized with 5 μg/mouse with Qβ-NS1-PEP immunogens or Qβ (VLPs alone, control, black lines) two times at 3-week intervals. Sera were collected and tested three weeks after the first immunization (day 21, red lines) and three weeks after the second immunization (day 42, blue lines). Sera were tested at various dilutions for binding to their cognate peptide using a peptide ELISAs.

In order to investigate the potential of these conserved regions to elicit antibodies that could bind to DENV NS1, we next generated synthetic peptides for each with a (Gly)_3_ Cys linker on the C-terminus of the peptide in order to facilitate chemical conjugation to bacteriophage Qβ VLPs. Chemical conjugation to Qβ VLPs was carried out as described in the methods and successful conjugation was confirmed by SDS-PAGE and Coomassie staining (Supplemental Figure S2). Mice were then immunized intramuscular with 5 μg/mouse without exogenous adjuvant two times at 3-week intervals, and blood was collected to assess antibody titers to cognate peptide (Figure 3, red lines). For the remainder of this report, we will refer to these vaccines collectively as Qβ-NS1-PEPs. Three weeks after the first immunization (day 21), we observed IgG antibody responses to all Qβ-NS1-PEPs except Qβ-112–122 and Qβ-154–170 (Figure 3, red lines). Three weeks after the second immunization (day 42), antibody titers of animals were tested again for antibody response to cognate peptides (Figure 3, blue lines). After second immunization, all Qβ-NS1-PEPs showed high-titer antibody responses, except Qβ-154–170, which showed negligible antibodies against cognate peptide. Animals were then sacrificed, and sera were collected for further studies.

### 3.4. Binding of Qβ-VLP-NS1-PEP Vaccine-Elicited Sera to Hexameric Recombinant NS1

**Figure 4:**
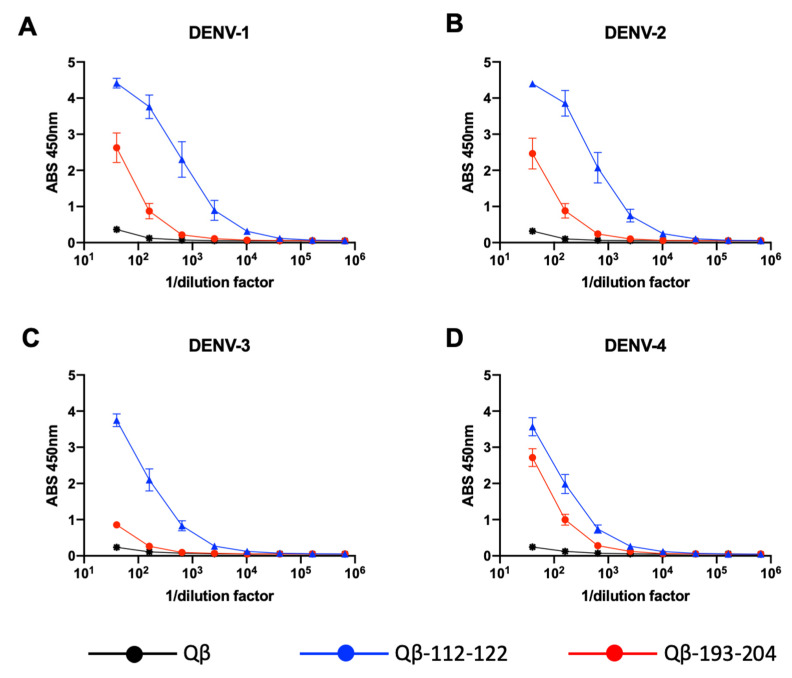
Qβ-VLP-NS1-PEP vaccine-elicited sera binding to soluble hexameric NS1. (**A**) DENV-1 NS1, (**B**) DENV-2 NS1, (**C**) DENV-3 NS1, (**D**) DENV-4 NS1.

Having found that the Qβ-VLP-NS1-PEPs are immunogenic in mice and elicit antibodies that bind to their cognate peptide by ELISA, we next investigated the ability of these immunogens to elicit antibodies that bind to DENV NS1 proteins. Using commercially available recombinant hexameric DENV NS1 proteins from all four DENV serotypes, we examined mouse immune sera for binding by ELISA. We first tested a pool of sera (3 weeks post second immunization) from each immunogen group to identify those immunogens that showed binding above Qβ VLP-immunized mouse sera (Supplemental Figure S3). From this, we identified sera from Qβ-112–122- and Qβ-193–204-immunized mice as capable of binding to DENV-NS1. To further investigate this, we performed ELISA with sera from the individual mice immunized with Qβ-112–122 and Qβ-193–204 (Figure 4) against NS1 from all four DENV serotypes. Here, we found Qβ-112–122 to be highly immunogenic to all four serotypes of DENV NS1. Qβ-193–204, however, was less immunogenic than Qβ-112–122, but still bound to DENV NS1 from all four serotypes. Interestingly, Qβ-112–122 and Qβ-193–204 were least immunogenic against NS1 from DENV-3.

### 3.5. Binding of Qβ-VLP-NS1-PEP Vaccine-Elicited Sera to DENV-Infected Cells

**Figure 5:**
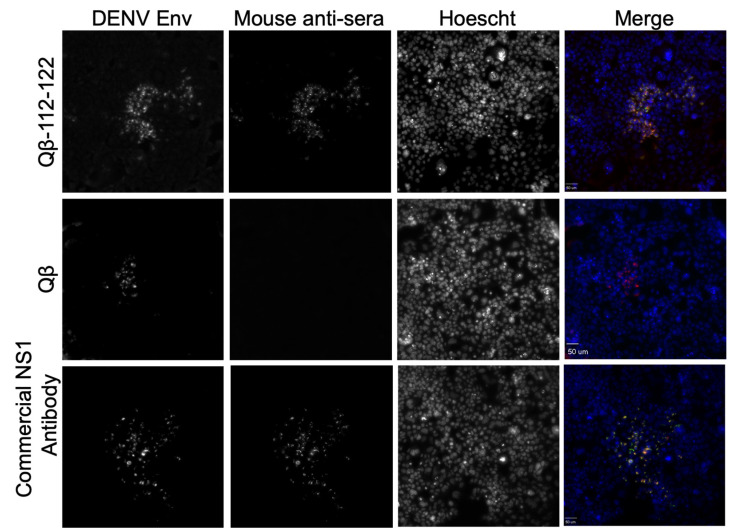
Binding of Qβ-VLP-NS1-PEP elicited antibodies to DENV-infected HEK293 cells. Cells were infected at a MOI of 100 for 72 h with DENV-2 NGC. Cells were then fixed with methanol and stained to detect DENV-infected cells (DENV Env, Red in Merge), sera from a Qβ-112–122-immunized mouse (Mouse anti-sera, Green in Merge) and nuclear staining with Hoechst (blue). A representative image generated with 1:320 dilution of sera from Qβ-112–122 or 1:40 dilution of sera from Qβ alone are shown here, along with a commercially available NS1 antibody as a positive control.

Our tests thus far have compared the ability of our sera antibodies to bind soluble hexameric NS1 protein from all four serotypes of DENV. However, NS1 in a dimeric form exists on the surface of DENV-infected cells as well as intracellularly. Here, we investigated whether or not antibodies from Qβ-VLP-NS1-PEPs bound to cell-associated NS1 in DENV-infected cells. Human embryonic kidney 293 (HEK 293) cells were infected with DENV from DENV-2 NGC at an MOI of 100 for 72 h. DENV-2 NGC was used since DENV-2 is the most commonly used serovar for laboratory studies. Infected cells were treated with a pan-flavivirus virus envelope antibody, 4G2, to identify infected cells. Additionally, sera from Qβ-VLP-NS1-PEP-immunized mice were added to the infected HEK 293 cells. Immunofluorescence microscopy was then performed to identify DENV-infected cells, as well as to identify whether our QB-VLP-NS1-PEP sera bound to the DENV-infected cells. Of our ten vaccine candidates, only sera from mice immunized against PEPs 112–122 bound to DENV-infected cells (Figure 5 and Supplemental Figure S4).

## 4. Discussion

DENV remains a severe threat to millions of people worldwide, yet a safe and effective vaccine that is useful for population-based vaccine campaigns has yet to be developed. A multitude of vaccine strategies targeting NS1 have been applied in order to solve this worldwide crisis, including protein, subunit peptide and DNA vaccines [12,17,18,21,22,23,24,25,26,27,28]. In addition to the current vaccine strategies, monoclonal and polyclonal sera have also been tested for efficacy of protection against severe DENV infections [12,17,18,19,20,52]. These methods have resulted in positive outcomes and survival of animals, indicating an important role for antibodies in the protection of DENV disease. However, while antibodies may aid in protection, antibodies that bind to the DENV envelope protein can also be detrimental and enhance infection through binding of host Fcγ receptors in a phenomenon coined Antibody-Dependent Enhancement (ADE) of infection. This supports the notion that antibody responses, while important, must be specific and safe to protect against DENV infection.

In our previous research, we utilized our deep sequence-coupled biopanning method (DSCB) to identify regions of the DENV genome in which patients made antibodies against [37,50]. The envelope (E) protein, specifically the Fusion Loop on the envelope protein, was a particularly immunodominant region; a result that has been observed in other studies [53,54,55,56,57]. Other immunodominant regions that have been identified are the wing domain (WD) portion of NS1 (aa 112–122), and the tail region of NS1 (aa 325–337), in which we identified a large number of patients making antibodies to these regions as well. These regions of the WD and NS1 tail are also highly conserved regions of the NS1 sequence, and we investigated these along with other conserved regions in this report.

We hypothesized that targeting conserved regions of NS1 would be a strategy for eliciting antibodies that would recognize NS1 from all four DENV serotypes. However, only aa 112–122 peptide was able to elicit antibodies that bound to NS1 in both the soluble and infected cell-associated forms. These results suggest that the other conserved regions of NS1 are not antigenically available for antibody binding. The WD of NS1 was the most successful vaccine that we identified (aa 112–122) due to the high level of antibody response in immunized mice, as well as the sera’s ability to bind soluble hexameric NS1 via ELISA and DENV-infected HEK293 cells. This was not altogether surprising based off of previous research that shows that the WD is a highly immunogenic epitope in all serotypes of DENV [39,40,41,42,43,44,45,46,47,48]. One obstacle of the WD is the amount of immunodominant antibodies and the uncertainty of the role it plays in DENV pathogenesis and whether these antibodies are indeed protective. However, Lai et al. has shown that by modifying the WD region, they are able to protect in some capacity, the severity of DENV disease in mice [18]. This modified WD epitope is a region, along with other modifications of the WD, that should be tested with our bacteriophage VLP technology. Additionally, an asparagine has been identified as a glycosylation site essential in the endocytosis and pathogenic function of NS1 [58]. These sites and others may be appropriate for additional investigation as vaccine targets.

In our previous research, the tail region of NS1 (aa 325–337) was highly selected by individuals who had been infected with their first DENV infection [50]. Additionally, antibodies from patients with secondary DENV infection had a binding profile similar to that of primary infected DENV patients, when sera were tested via ELISA [50]. However, our results provided in the current study show that the immunization against the NS1 tail region (aa 325–337) elicited high antibody titers to the cognate peptide, but these antibodies insufficiently bound to soluble NS1 and DENV-infected cells. This may be due to the fact that our Qβ VLP vaccines are displaying the peptide epitopes in a linear fashion. Additionally, our peptide ELISAs test the binding of sera antibodies to the linear peptides on the surface of the plate. However, in our previous work the peptide ELISAs testing human sera against the NS1 tail peptide were testing sera antibodies in the same way [50]. This indicates that humans naturally infected with primary and secondary DENV infections made antibodies that were capable of binding to the linear form of the NS1 tail epitope. One explanation for this observation may be location of this NS1 tail region on the NS1 dimer. Based off of our structural analysis in Figure 2, the surface-exposed regions of NS1 tail region (red) seems to be on the outer edges of the NS1 dimer. However, our studies here are also testing antibody binding to the hexameric form of NS1. These important binding sites may be hidden in the hexameric form of soluble NS1.

The benefit of our bacteriophage VLP platform, compared to technologies such as immunization with whole protein, is the ability to make specific antibodies to selected epitopes. This specificity allows control of the antibody profile such that it can be functional, yet safe by avoiding pathogenic antibodies during DENV infection. Additionally, previous work in our lab and others has shown that bacteriophage VLP-based vaccines induce high-titer, long-lasting antibody responses that do not require the addition of exogenous adjuvant, making our system relatively fast, easy, and safe to develop [31,32,59,60]. Our results show that our VLP-based NS1 vaccines have the capacity to high-titer antibodies against specific DENV NS1 epitopes and these antibodies bind to both hexameric soluble NS1 and cell-associated NS1. Further analysis of the functional characteristics and abilities of these vaccine candidates will be assessed in future studies. Functional assays such as protection against endothelial barrier disruption, peripheral blood mononuclear cell activation, and the protective capacity of these vaccines in an animal model of NS1-mediated pathogenesis will be investigated in future studies. Furthermore, other epitopes of the DENV NS1 protein could be investigated to identify other regions that may be protective in severe DENV disease.

## Data Availability

The data presented in this study are available in the article.

## Supplementary Materials

The following are available online at https://www.mdpi.com/article/10.3390/vaccines9070726/s1; Figure S1: NS1 protein epitopes; Figure S2: SDS-PAGE confirmation of peptide conjugation to Qβ VLPs; Figure S3: Binding of Qβ-NS1-PEP sera to soluble hexameric NS1 protein; Figure S4: Binding of Qβ-NS1-PEP sera to DENV-infected HEK293 cells.
