# Supplementary material for: Development of Bacteriophage Virus-Like Particle Vaccines Displaying Conserved Epitopes of Dengue Virus Non-Structural Protein 1
Source: Vaccines (Basel). 2021 Jul 2;9(7):726. doi: 10.3390/vaccines9070726 (PMC8310087; doi:10.3390/vaccines9070726)
Supplement: Supplementary file 1 [file vaccines-09-00726-s001.zip › vaccines-1269211-supplementary.pptx]

## Slide 1
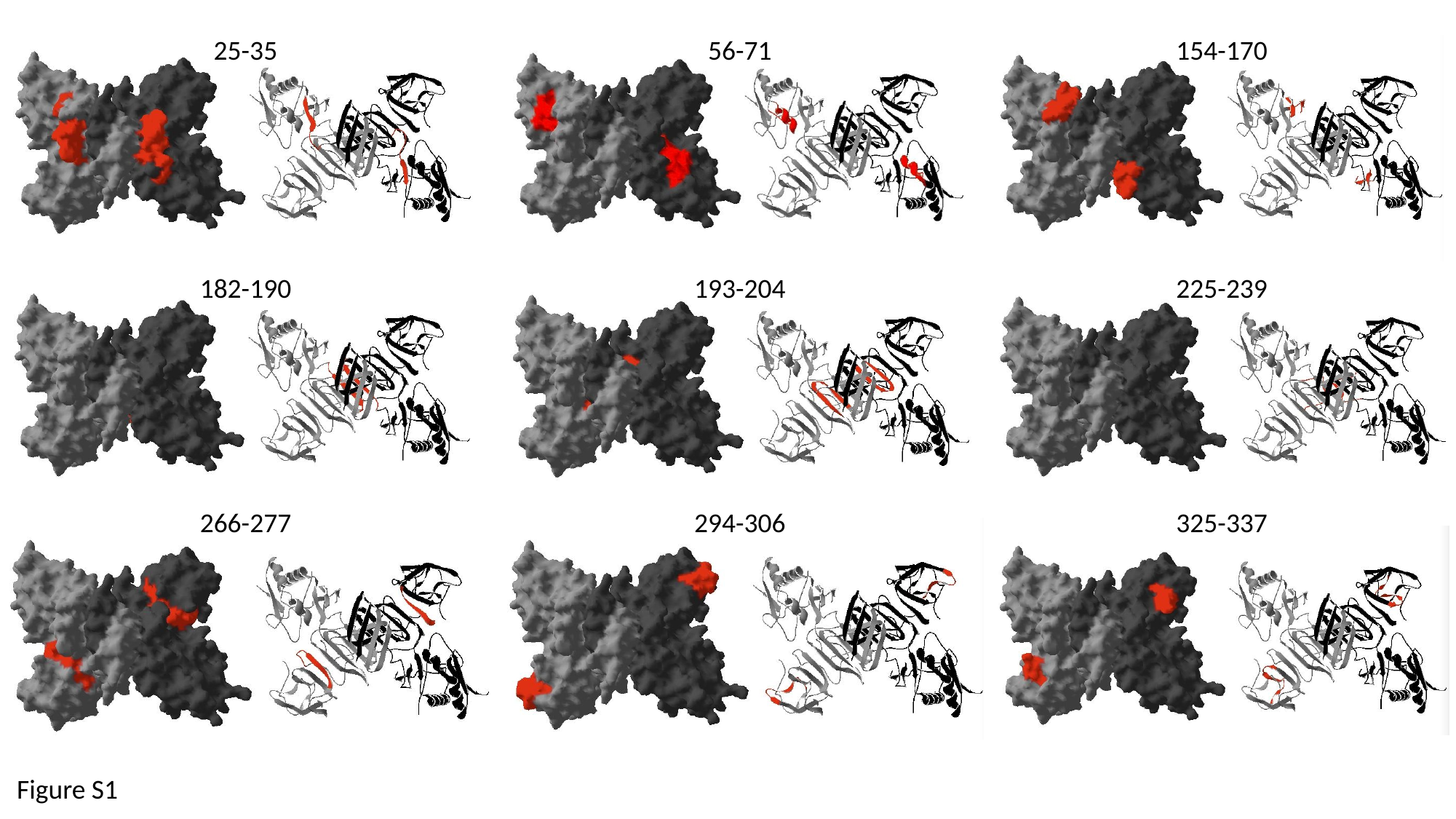

25-35
56-71
154-170
182-190
193-204
225-239
266-277
294-306
325-337
Figure S1

## Slide 2
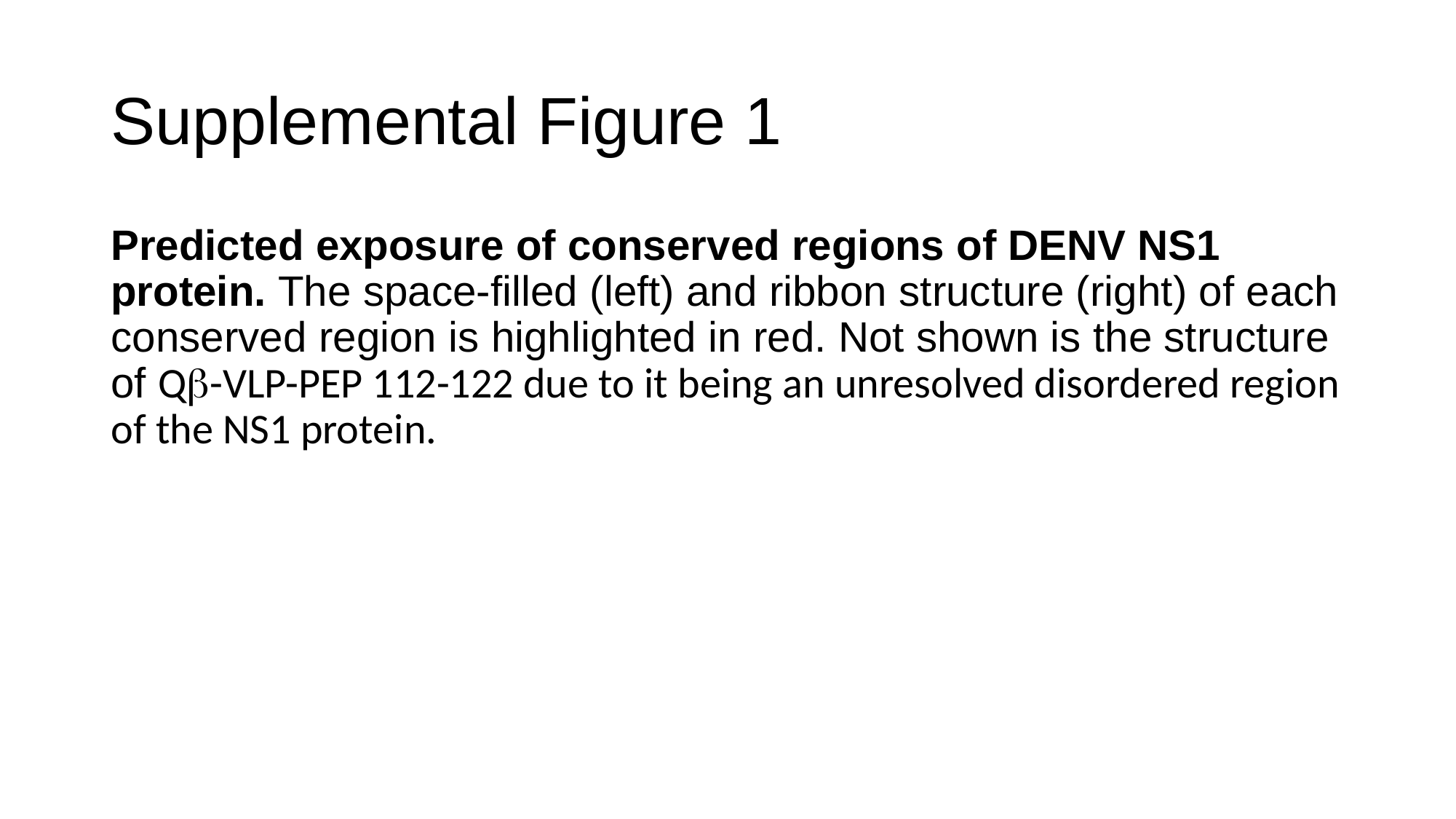

# Supplemental Figure 1
Predicted exposure of conserved regions of DENV NS1 protein. The space-filled (left) and ribbon structure (right) of each conserved region is highlighted in red. Not shown is the structure of Qβ-VLP-PEP 112-122 due to it being an unresolved disordered region of the NS1 protein.

## Slide 3
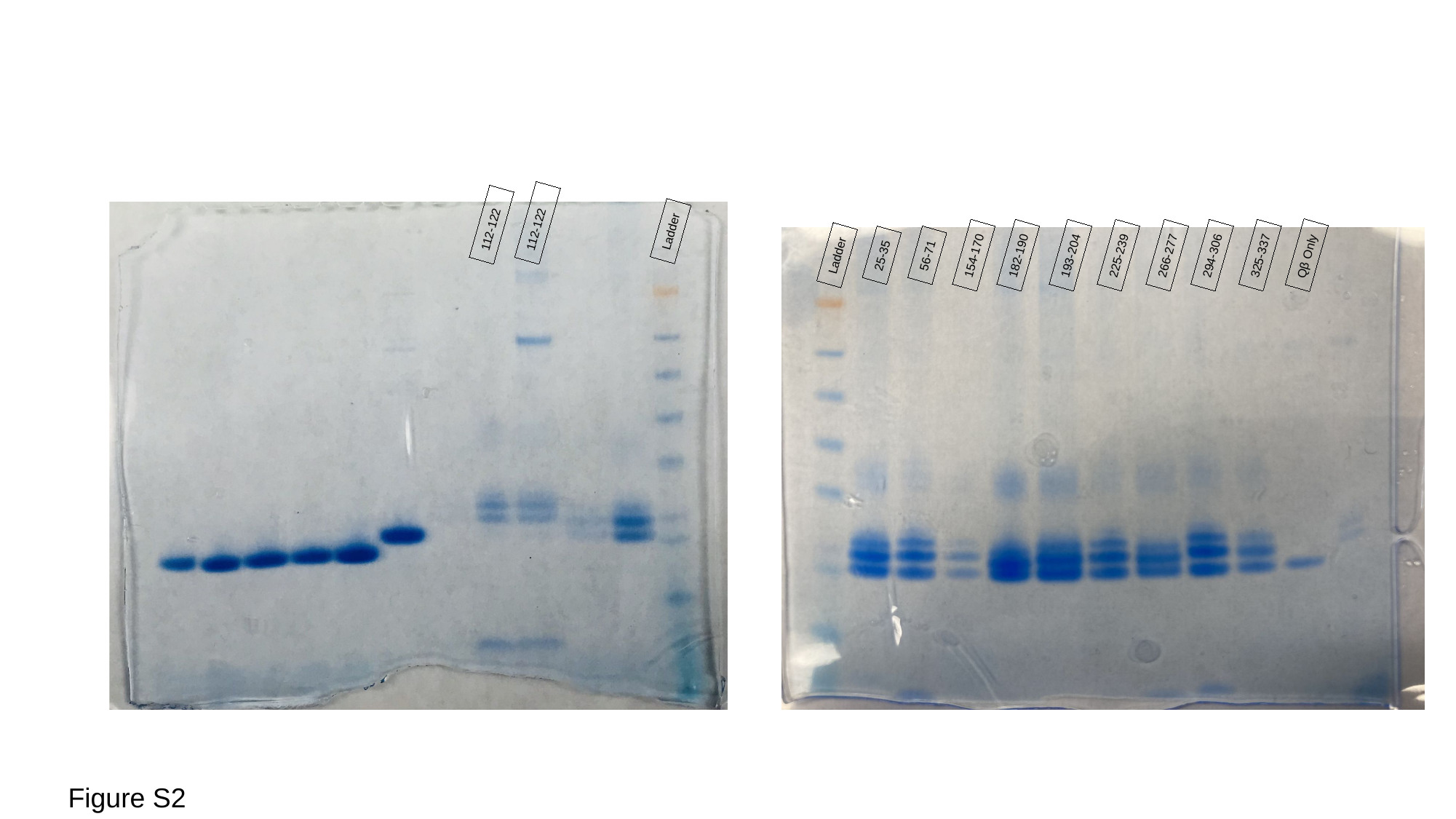

112-122
112-122
Ladder
Ladder
25-35
56-71
154-170
182-190
193-204
225-239
266-277
294-306
325-337
Qβ Only
Figure S2

## Slide 4
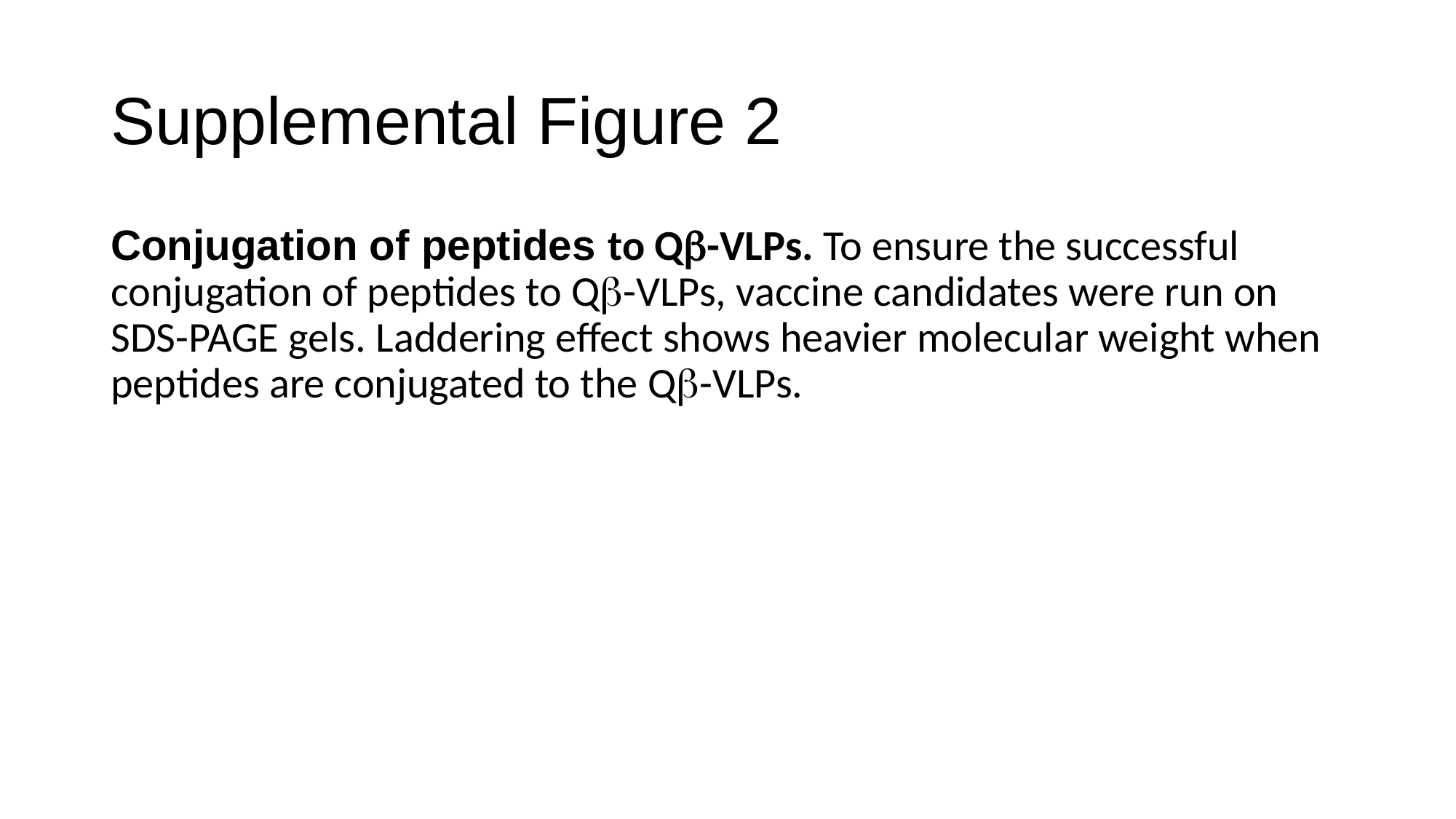

# Supplemental Figure 2
Conjugation of peptides to Qβ-VLPs. To ensure the successful conjugation of peptides to Qβ-VLPs, vaccine candidates were run on SDS-PAGE gels. Laddering effect shows heavier molecular weight when peptides are conjugated to the Qβ-VLPs.

## Slide 5
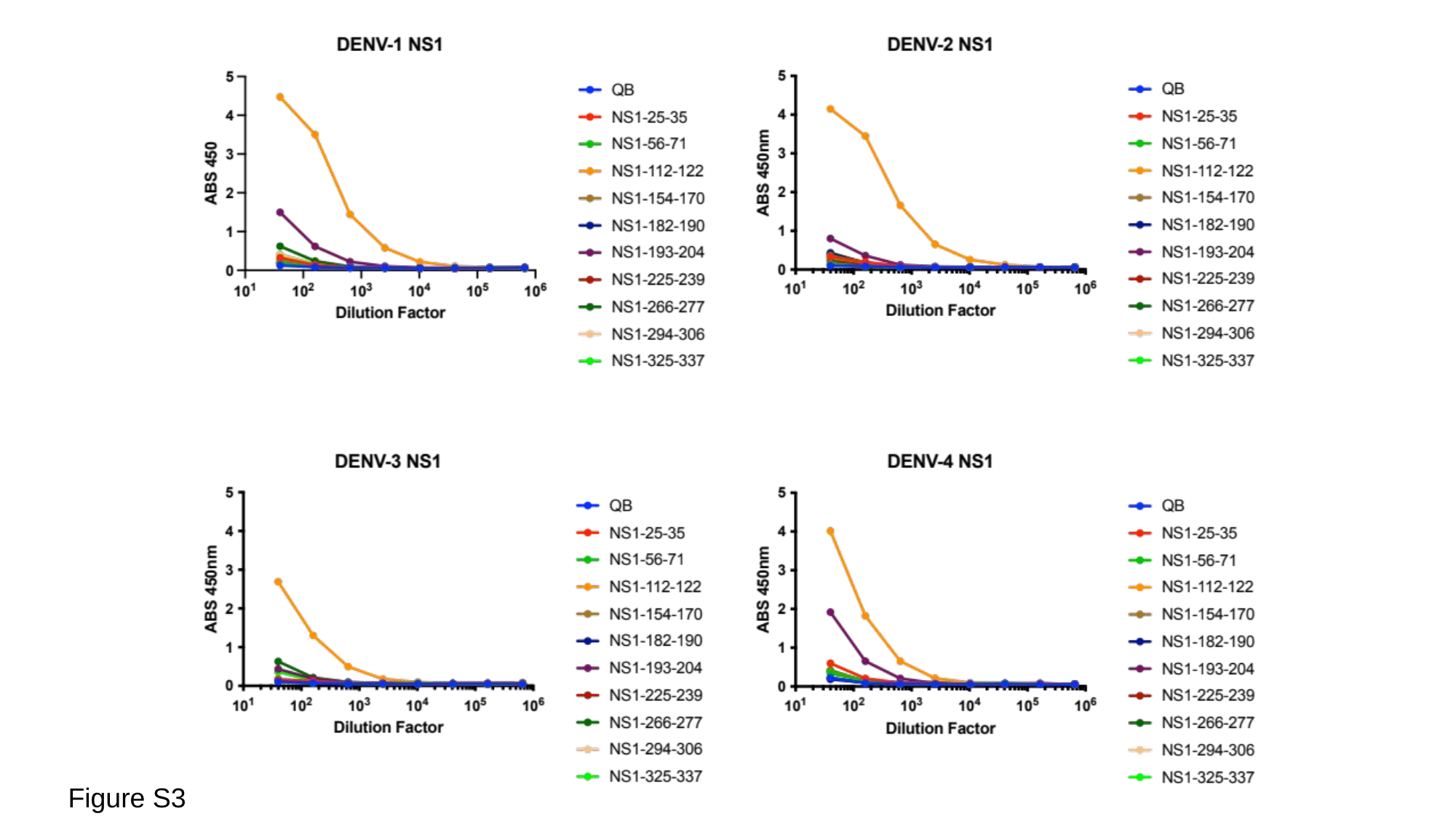

Figure S3

## Slide 6
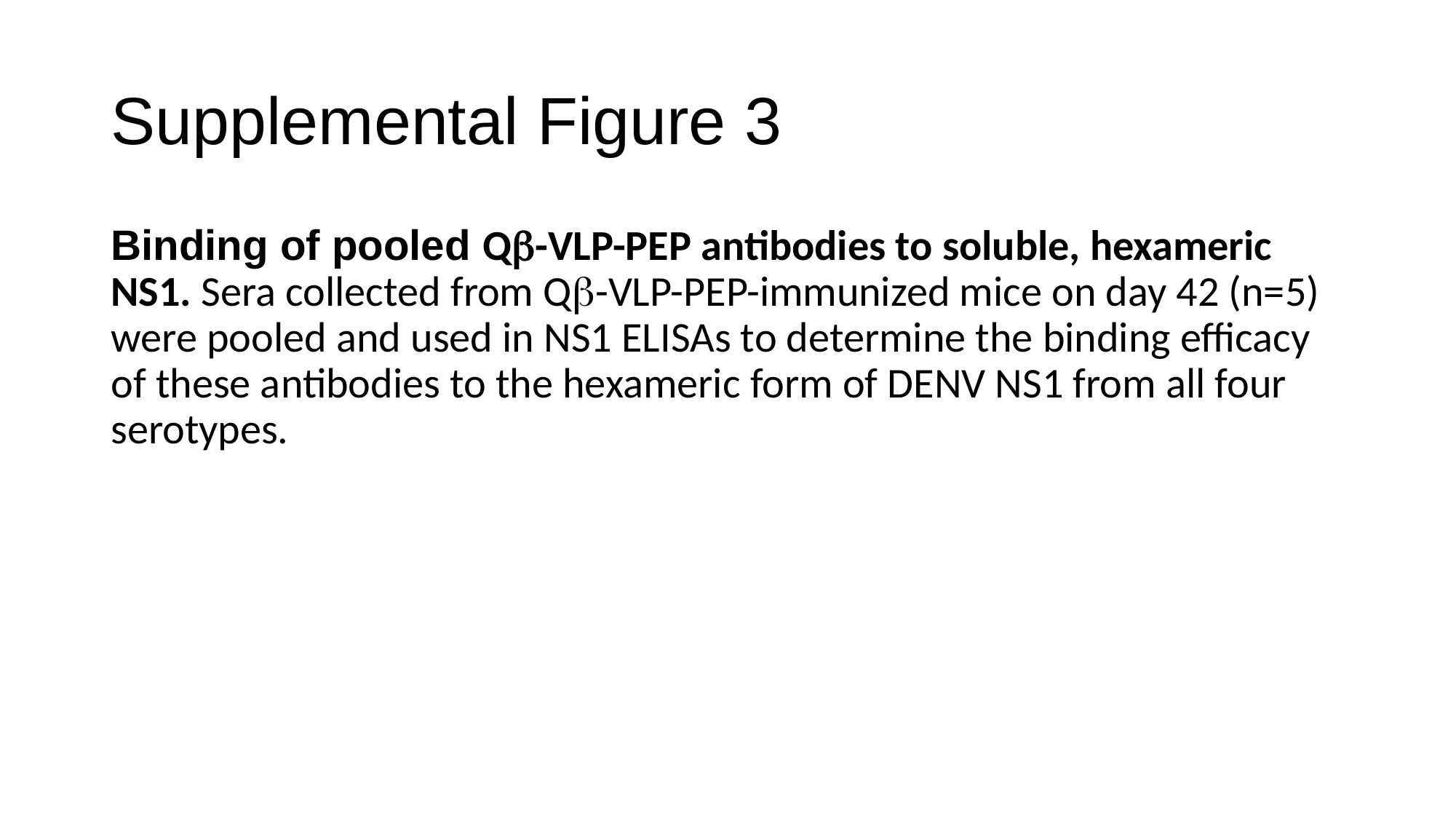

# Supplemental Figure 3
Binding of pooled Qβ-VLP-PEP antibodies to soluble, hexameric NS1. Sera collected from Qβ-VLP-PEP-immunized mice on day 42 (n=5) were pooled and used in NS1 ELISAs to determine the binding efficacy of these antibodies to the hexameric form of DENV NS1 from all four serotypes.

## Slide 7
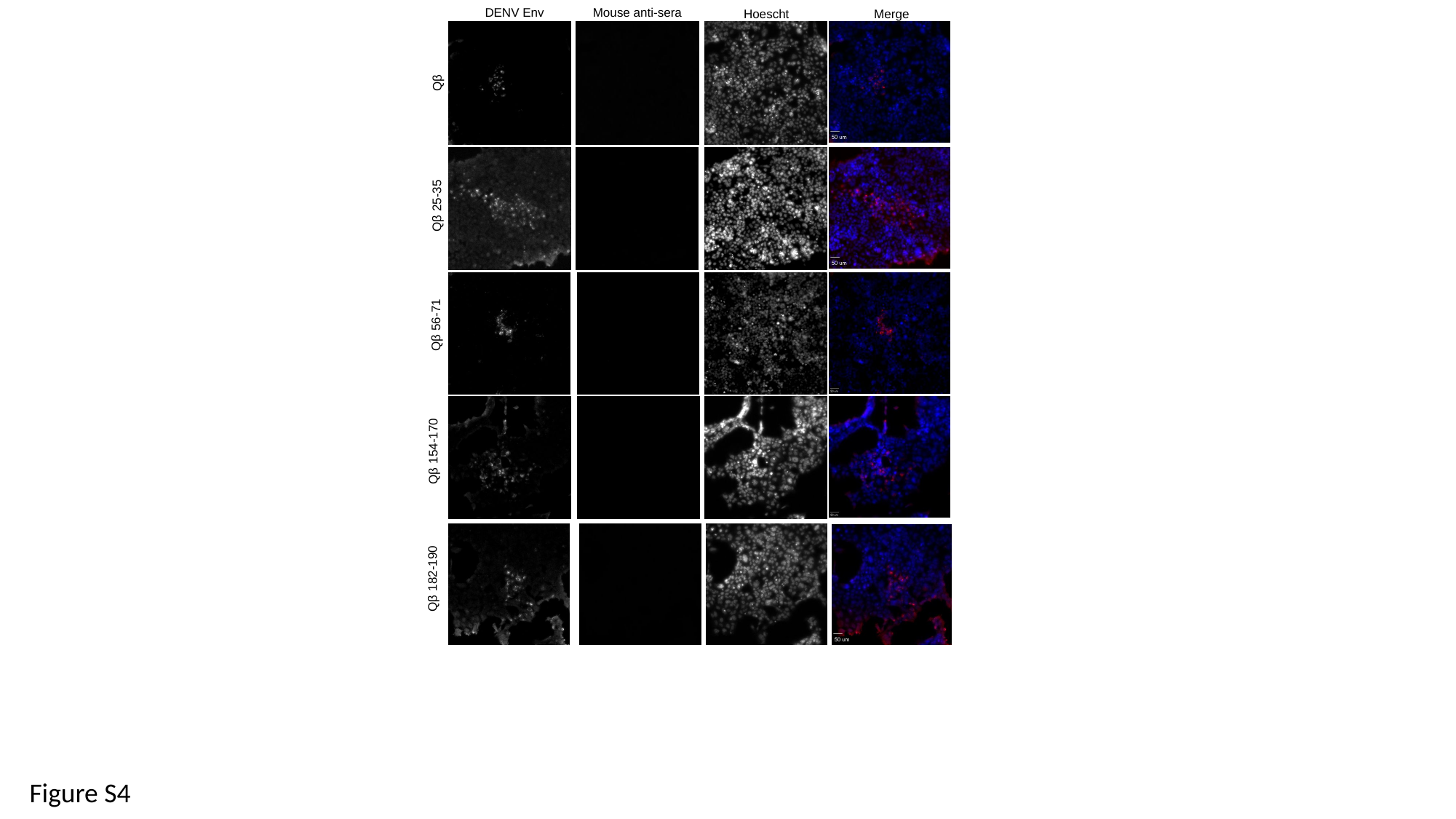

DENV Env
Mouse anti-sera
Merge
Hoescht
Qβ
Qβ 25-35
Qβ 56-71
Qβ 154-170
Qβ 182-190
Figure S4

## Slide 8
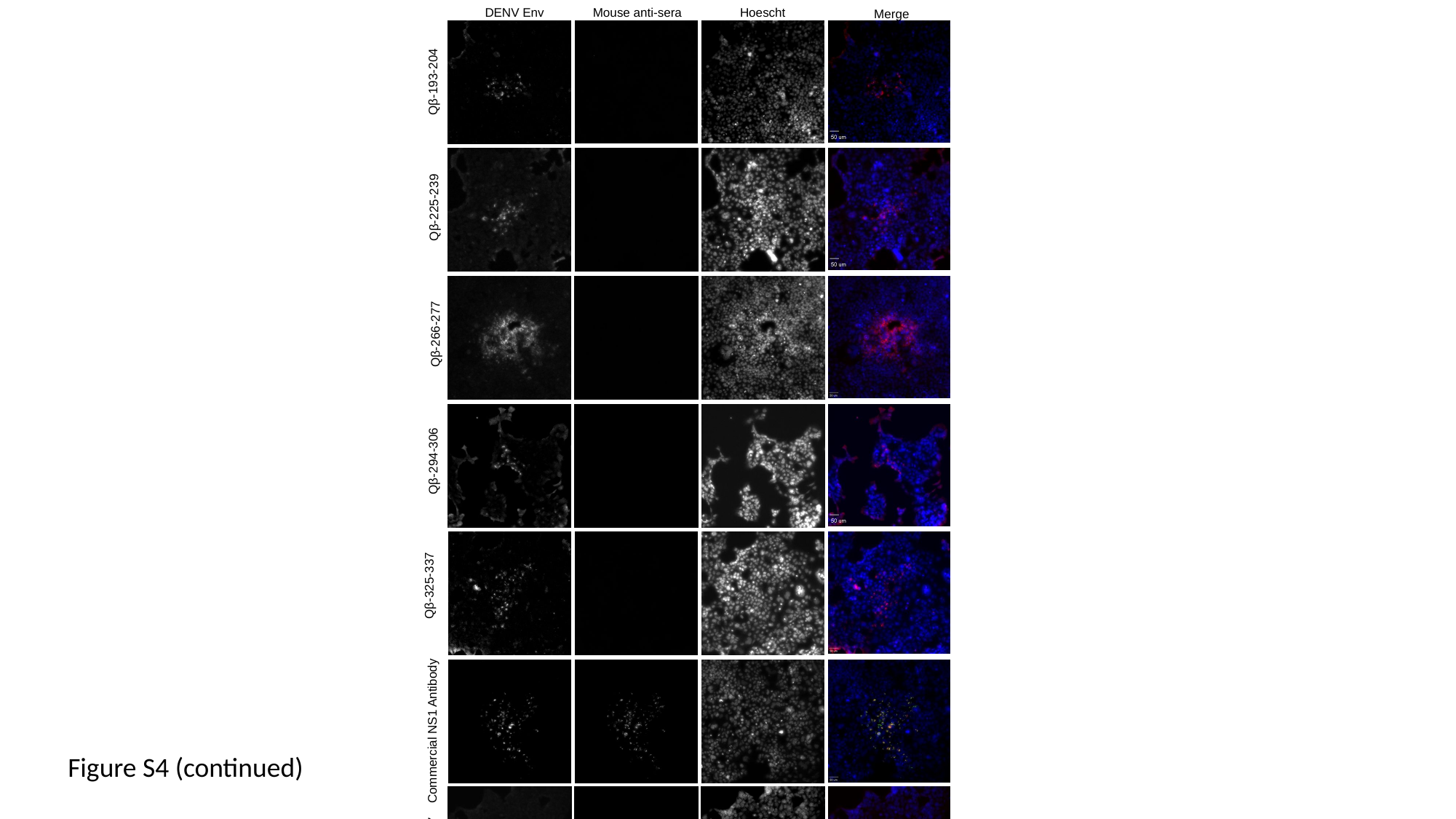

DENV Env
Hoescht
Mouse anti-sera
Merge
Qβ-193-204
Qβ-225-239
Qβ-266-277
Qβ-294-306
Qβ-325-337
Commercial NS1 Antibody
Figure S4 (continued)
Secondary 647
Secondary 488

## Slide 9
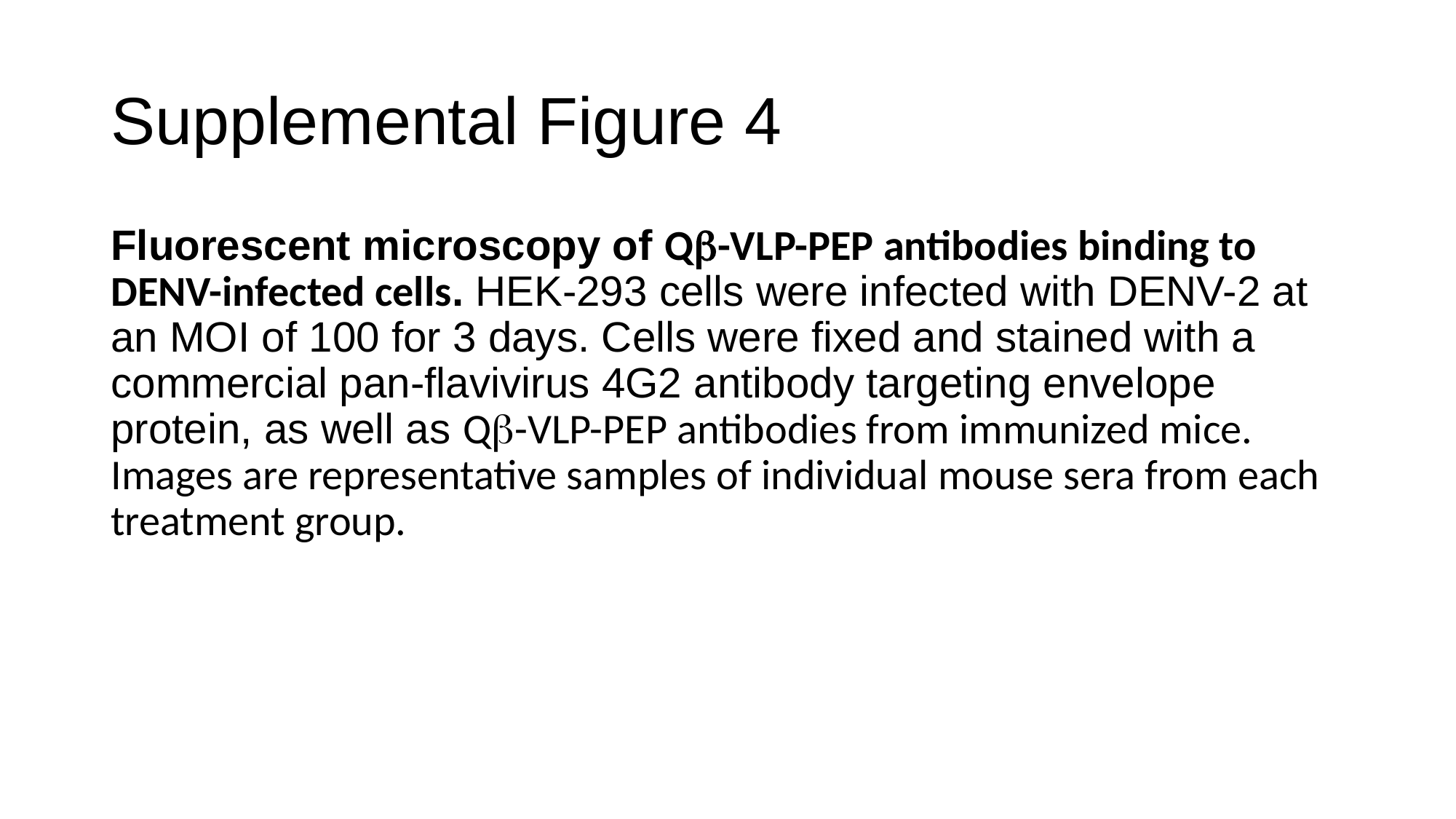

# Supplemental Figure 4
Fluorescent microscopy of Qβ-VLP-PEP antibodies binding to DENV-infected cells. HEK-293 cells were infected with DENV-2 at an MOI of 100 for 3 days. Cells were fixed and stained with a commercial pan-flavivirus 4G2 antibody targeting envelope protein, as well as Qβ-VLP-PEP antibodies from immunized mice. Images are representative samples of individual mouse sera from each treatment group.
